# Application of an Ultrasensitive NGS-Based Blood Test for the Diagnosis of Early-Stage Lung Cancer: Sensitivity, a Hurdle Still Difficult to Overcome

**DOI:** 10.3390/cancers14082031

**Published:** 2022-04-18

**Authors:** Malaïka Van der Linden, Bram Van Gaever, Lennart Raman, Karim Vermaelen, Ingel Demedts, Veerle Surmont, Ulrike Himpe, Yolande Lievens, Liesbeth Ferdinande, Franceska Dedeurwaerdere, Joni Van der Meulen, Kathleen Claes, Björn Menten, Jo Van Dorpe

**Affiliations:** 1 Department of Diagnostic Sciences, Ghent University, 9000 Ghent, Belgium; malaika.vanderlinden@ugent.be (M.V.d.L.); bram.vangaever@ugent.be (B.V.G.); lennart.raman@gmail.com (L.R.); liesbeth.ferdinande@ugent.be (L.F.); 2 Cancer Research Institute Ghent, 9000 Ghent, Belgium; karim.vermaelen@ugent.be (K.V.); yolande.lievens@ugent.be (Y.L.); joni.vandermeulen@ugent.be (J.V.d.M.); kathleen.claes@ugent.be (K.C.); 3 Department of Pathology, Ghent University Hospital, 9000 Ghent, Belgium; 4 Department of Pulmonary Medicine, Ghent University Hospital, 9000 Ghent, Belgium; veerle.surmont@ugent.be; 5 Department of Internal Medicine and Pediatrics, Ghent University, 9000 Ghent, Belgium; 6 Department of Pulmonary Medicine, AZ Delta, 8800 Roeselare, Belgium; ingel.demedts@azdelta.be (I.D.); ulrike.himpe@azdelta.be (U.H.); 7 Department of Radiation Oncology, Ghent University Hospital, 9000 Ghent, Belgium; 8 Department of Human Structure and Repair, Ghent University, 9000 Ghent, Belgium; 9 Department of Pathology, AZ Delta, 8800 Roeselare, Belgium; franceska.dedeurwaerdere@azdelta.be; 10 Center for Medical Genetics, Ghent University Hospital, 9000 Ghent, Belgium; bjorn.menten@ugent.be; 11 Department of Biomolecular Medicine, Ghent University, 9000 Ghent, Belgium

**Keywords:** early-stage non-small cell lung cancer, liquid biopsy, cell-free DNA, circulating tumor DNA, next-generation sequencing, somatic mutations, cancer detection

## Abstract

**Simple Summary:**

Currently, an accurate diagnosis of lung cancer relies on the microscopic examination of tissue biopsies. These samples can, however, only be obtained by invasive procedures. The aim of our study was to evaluate the use of a liquid biopsy for early-stage lung cancer detection in patients with a lung lesion on imaging. This approach would be particularly relevant for suspected lung lesions that are difficult to reach for a tissue-based diagnosis. Despite technical improvements for the use of liquid biopsy-based cell-free DNA analysis, its application for the detection of early-stage lung cancer is currently limited by sensitivity and a biological background of somatic variants.

**Abstract:**

Diagnosis of lung cancer requires histological examination of a tissue sample, which in turn requires an invasive procedure that cannot always be obtained. Circulating tumor DNA can be reliably detected in blood samples of advanced-stage lung cancer patients and might also be a minimally invasive alternative for early-stage lung cancer detection. We wanted to explore the potential of targeted deep sequencing as a test for the diagnosis of early-stage lung cancer in combination with imaging. Mutation detection on cell-free DNA from pretreatment plasma samples of 51 patients with operable non-small cell lung cancer was performed and results were compared with 12 control patients undergoing surgery for a non-malignant lung lesion. By using a variant allele frequency threshold of 1%, somatic variants were detected in 23.5% of patients with a median variant allele fraction of 3.65%. By using this threshold, we could almost perfectly discriminate early-stage lung cancer patients from controls. Our study results are discussed in the light of those from other studies. Notwithstanding the potential of today’s techniques for the use of liquid biopsy-based cell-free DNA analysis, sensitivity of this application for early-stage lung cancer detection is currently limited by a biological background of somatic variants with low variant allele fraction.

## 1. Introduction

Currently, an accurate diagnosis of lung cancer relies on the histological examination of biopsied malignant tissue [1]. This material can, however, only be obtained by invasive techniques. For central tumors or tumors with metastases in perihilar or mediastinal lymph nodes, bronchoscopy and endobronchial ultrasound with transbronchial needle aspiration can be used. Peripheral lesions are more difficult to biopsy and only some of these lesions can be sampled by a percutaneous computed tomography-guided transthoracic lung biopsy, a procedure sometimes complicated by a clinically relevant pneumothorax. These investigations are sometimes not possible in older or debilitated patients, or patients with inaccessible lesions, and may involve unacceptable medical risk in patients having substantial comorbidity. Hence, some patients proceed to surgery or stereotactic body radiotherapy without a proper diagnosis [2,3,4,5,6]. It has been shown that in cancer patients, tumor-derived DNA can be present in blood, circulating as a fraction of cell-free DNA (cfDNA), called circulating tumor DNA (ctDNA). Hence, detection and analysis of this ctDNA through a so-called liquid biopsy offers a minimally invasive alternative for cancer detection [7]. Lung cancers harbor numerous genetic alterations that comprise single nucleotide variants (SNVs), insertions and deletions (indels), as well as copy number variations (CNVs) and rearrangements. We have previously shown that ctDNA can be detected in the blood of advanced-stage lung cancer patients by using shallow whole-genome sequencing of cfDNA for CNV detection and that it can be used for subtyping of small versus non-small cell lung cancer [8]. However, detection of early-stage lung cancer is limited by low ctDNA fractions down to 0.008% for lung tumors with a volume of 1 cm^3^ [9] and thus requires ultrasensitive and ultra-deep sequencing. Cancer personalized profiling by deep sequencing (CAPP-Seq) has been published as a cfDNA analysis method with potential for early-stage lung cancer detection, but also other sensitive approaches have recently been published [9,10,11]. The CAPP-Seq library preparation protocol is optimized for low cfDNA input and uses a panel of genes recurrently mutated in lung cancer [12]. The method was further improved with integrated digital error suppression (iDES) by using unique molecular identifiers for recovery of cfDNA molecules and in silico removal of stereotypical artefacts [13]. Recently, Chabon et al. developed the machine-learning method “Lung Cancer Likelihood in Plasma (Lung- CLiP)”, integrating cfDNA analysis-based features derived from non-small cell lung cancer (NSCLC) cases and matched controls [14].

Here, we present the evaluation of a commercially available liquid biopsy assay, the AVENIO ctDNA Surveillance Kit (Roche, Pleasanton), based on iDES-enhanced CAPP-Seq, for the diagnosis of lung lesions that appeared suspect on imaging. Pre-operative cfDNA from 51 patients with operable NSCLC and 12 control patients with an operated non-neoplastic lung lesion was analyzed and compared.

## 2. Materials and Methods

### 2.1. Study Population

This study describes the prospective analysis of plasma samples from 51 patients with early-stage/operable NSCLC with a lesion suspect on imaging who underwent a surgical resection. Additionally, 12 control patients with a non-neoplastic (non-malignant) lung lesion, such as amyloidosis, pleurisy, pneumonia, silicosis, and vasculitis, were included as negative controls to evaluate specificity. From January 2016 until January 2020, patients were recruited at Ghent University Hospital and AZ Delta Roeselare. The median age was 66 years (range 36–83 years) for the patients and 64 years (range 57–74 years) for the controls. Although patient inclusion was random, the final study population was enriched for (active) smokers; 90.2% (46/51) of the patients and 75% (9/12) of the controls were active smokers or had a history of smoking. For subtyping of NSCLCs and staging, the 2016 World Health Organization’s and International Union Against Cancer 8th edition TNM criteria were used. For lung adenocarcinomas, standard molecular analysis was performed for *EGFR* mutations by using an *EGFR* mutation test (Ghent University Hospital only) and/or next-generation sequencing (NGS). Patients predominantly had stage I disease (49% or 25/51). Adenocarcinoma (62.7% or 32/51) and T2a (T > 3 cm but ≤ 4 cm in greatest dimension; 21.6% or 11/51) were the most frequent subtype and stage. In tumor tissue from four patients an *EGFR* (C01) or a *KRAS* (C13, C14 and C32) mutation was detected. Patient’s main clinical characteristics, including age, histology, tumor size, TNM classification, disease stage, and smoking status are listed in Supplementary Tables S1 and S2 and summarized in Table 1.

### 2.2. Sample Collection and Processing

For both patients and controls, two blood samples were collected before surgery. Blood was collected in cell-free DNA BCT tubes (Streck, La Vista, NE, USA) or PAXgene blood ccfDNA tubes (PreAnalytiX, Hombrechtikon, Switzerland) for cfDNA-analysis. Within 24 h of collection, plasma was separated by one centrifugation step at 1900× *g* for 15 min at room temperature (PAXgene) or two centrifugation steps, first at 1600× *g* for 10 min at 4 °C and second at 16,000× *g* for 10 min at 4 °C (Cell-Free DNA BCT), and stored at −80 °C upon use. cfDNA was isolated from 4 mL of plasma (one blood tube) by using the Maxwell RSC ccfDNA plasma kit (Promega, Madison, WI, USA) and accompanying instructions, and quantified by using a fluorescence dsDNA high sensitivity assay (Thermo Fisher Scientific, Waltham, MA, USA). For 19 patients and four controls, an additional 4 mL plasma sample (second blood tube) was used because the yield of the first isolation was too low to proceed with library preparation. Six patients and four controls were excluded from the analysis due to insufficient cfDNA yield (five patients and two controls) or the presence of high molecular weight DNA (one patient and two controls).

#### 2.2.1. AVENIO ctDNA Surveillance Assay

Roche’s AVENIO ctDNA surveillance kit was evaluated for the diagnosis of early-stage lung cancer. This assay makes use of a cfDNA-optimized library preparation protocol, including molecular barcoding, and targeted sequencing of a ~200 kb gene panel, covering 471 frequently mutated regions across 197 genes, associated with lung and colorectal cancer, and allows one to screen for SNVs, indels, rearrangements, and amplifications.

Sequencing library samples were prepared according to the manufacturer’s instructions, starting from 10 to 50 ng of input DNA (median of 16 ng in patients and 15 ng in controls). The maximum possible DNA input was used. The library samples were sequenced on a HiSeq 3000 or NextSeq 500 Illumina platform. Libraries were pooled to generate 60 million paired-end reads per sample, resulting in a median unique depth of 2818× and 2885× for patients and controls, respectively. Four patients and two controls were additionally excluded from the analysis due to low library yield. Sample metrics are listed in Supplementary Tables S3 (patients) and S4 (controls).

Sequencing data was analyzed by using the AVENIO oncology analysis software (version 1.1.0 and 2.0.0; Roche). This software provides a sequencing quality report with key metrics such as sequencing depth, number of reads, on-target rate, coverage uniformity, and error rate to confirm quality at different stages in the workflow, and a variant report. Default filtering keeps known somatic variants thus variants seen in the Catalogue of Somatic Mutations in Cancer (COSMIC) and The Cancer Genome Atlas (TCGA) databases, and variants in the Loci of Interest (LOI) list, regardless of filter settings, and removes known germline variants, thus everything in the Exome Aggregation Consortium (ExAC) or 1000 Genomes databases with allele frequency (AF) > 0.1%, or in the Single Nucleotide Polymorphism database (dbSNP) Common. Detection of indels, rearrangements and amplifications are limited to a pre-specified list of variants. We further filtered out (1) synonymous variants; (2) variants for which the highest subpopulation frequency in the Genome Aggregation Database (gnomAD) is >0.2% (Custom Filtering 1–2); (3) possible germline variants (AF > 40%); and (4) variants for which the AF is <1% (custom filtering 3–4).

#### 2.2.2. Variant Confirmation

For the first 12 patients (C01-C12), variant confirmation was performed on the formalin-fixed, paraffin-embedded (FFPE) surgically resected tissue or white blood cell (WBC)-derived genomic DNA (gDNA), when it was probably a germline or clonal hematopoiesis-derived variant. DNA was extracted from FFPE tumor (FFPET) tissue sections by using the QIAamp DNA FFPE tissue kit and deparaffinization solution (Qiagen, Hilden, Germany), and quantified by using a fluorescence dsDNA broad range assay (Thermo Fisher Scientific). Blood collected before surgery in a 3 mL, small EDTA tube was used for gDNA isolation. gDNA was isolated by using the ReliaPrep large volume HT gDNA isolation system (Promega) or QIAamp DNA blood mini kit (Qiagen), and quantified by using the UV/Vis DropSense system (Trinean, Ghent, Belgium). For both, FFPET-derived DNA and gDNA samples, a singleplex polymerase chain reaction (PCR)-based targeted amplicon NGS approach was used as previously described [15]. Briefly, following a universal PCR protocol, samples were pooled with other PCR products and prepared for sequencing by using the NexteraXT (Illumina, San Diego, CA, USA) protocol. Samples were sequenced on an Illumina MiSeq instrument (2 × 150 cycles) and NGS data were analyzed by using a variant AF threshold of 2% or 1% for FFPET or gDNA samples, respectively. For two FFPET samples (C11 and C32), sequencing was performed after capture-based enrichment for a specific gene panel.

For the following 32 patients (C13–C44), the FFPE surgically resected tissue was analyzed by using Roche’s AVENIO Tumor Tissue Surveillance Kit. DNA isolation and sequencing library preparation were performed according to the manufacturer’s user guide. The libraries were sequenced with the Illumina HiSeq 3000 instrument. For eight patients, FFPET analysis was not possible due to unavailability of material (*n* = 2), low DNA quality (*n* = 2), low DNA yield (*n* = 2), or library preparation error (*n* = 2). Sequencing data was analyzed by using the AVENIO oncology analysis software (version 2.0.0; Roche) as described for cfDNA samples except that we further filtered out (custom filtering) (1) synonymous variants; (2) variants for which the highest subpopulation frequency in gnomAD is >0.1%; (3) variants for which the AF is <5%, except known hotspot variants (defined by the SNV LOI list) with an AF between 2% and 5%, and variant and unique depth of 10 and 300, respectively; and (4) possible germline variants (in TCGA and AF is >40%). Sample metrics are listed in Supplementary Table S5. Possible germline and clonal hematopoiesis-derived variants in cfDNA were confirmed on gDNA as described above.

For the last seven patients (C45–51), no variant confirmation was performed.

Only possible germline variants were confirmed on WBC gDNA of control patients.

### 2.3. Statistical Analysis

Statistical tests were performed in R, and include Wilcoxon singed-rank test and calculation of the Pearson correlation coefficient.

The sensitivity and specificity of this approach of somatic variant detection for the diagnosis of early-stage lung cancer was established by comparing the variant reports with the histological diagnosis.

## 3. Results

To evaluate the use of a liquid biopsy for lung cancer detection, we analyzed the cfDNA of 51 patients with early-stage NSCLC and 12 control patients by using a commercially available liquid biopsy assay, AVENIO ctDNA Surveillance Kit (Roche, Pleasanton).

**Figure A1:**
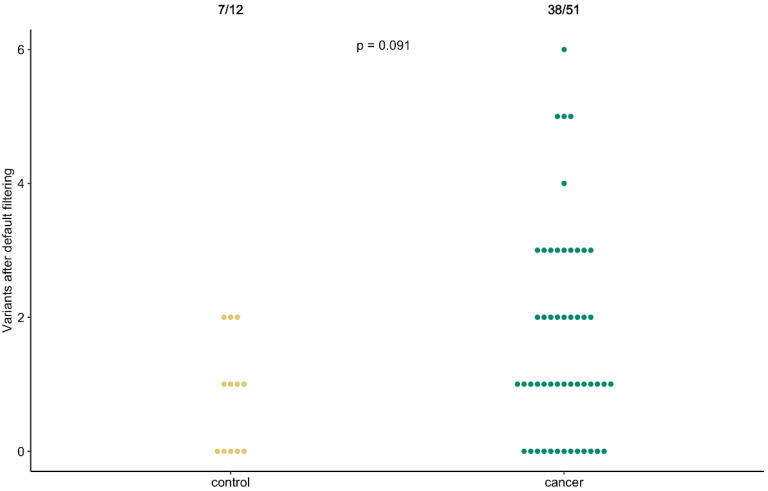
Default variant filtering cannot significantly distinguish patients from controls. Overall variant detection in control (yellow) and cancer (green) patients using default filtering. Beeswarm plots represent the number of somatic variants reported for a sample. The detection rate and Pearson correlation coefficient are given at the top.

Default variant filtering resulted in variant detection in 74.5% (38/51) of NSCLC patient and 58.3% (7/12) of control cfDNA samples, with a median of one variant per sample in each cohort (and a maximum of six or two variants, respectively), and could not significantly distinguish patients from controls (*p* = 0.091; Appendix A, Figure A1, and Supplementary Tables S6 (patients) and S7 (controls)).

**Figure 1:**
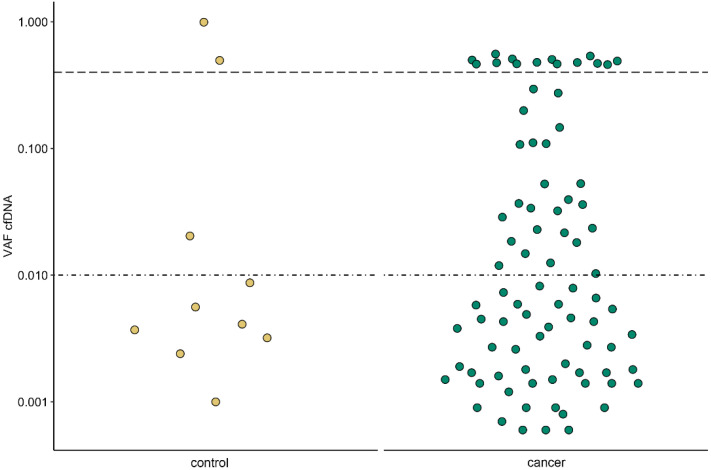
Cell-free DNA (cfDNA) variant allele fractions (AF) observed in control (left/yellow) and cancer patients (right/green) by using default filtering. The variant AF thresholds used for custom filtering are shown as horizontal lines at 1% and 40% variant AF.

**Figure 2:**
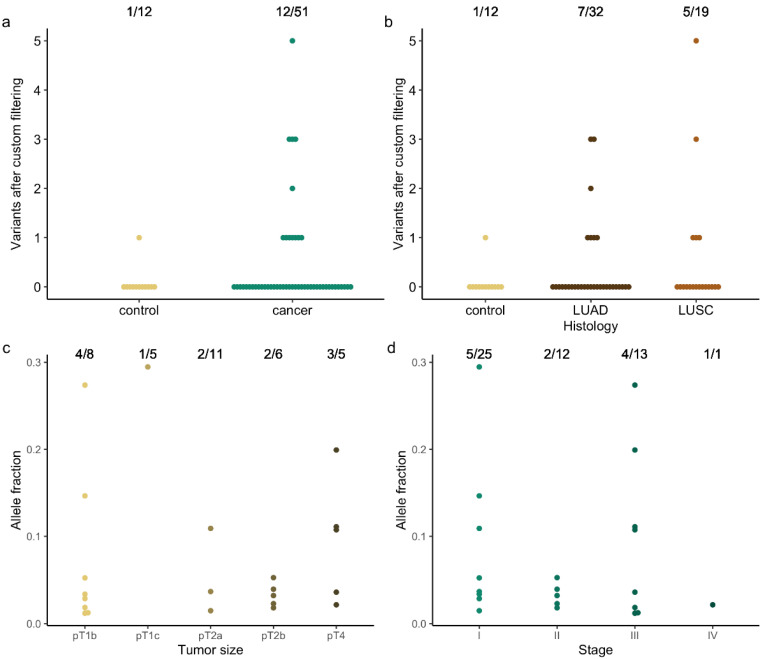
Somatic variant detection in control and cancer patients (**a**,**b**) and correlation with disease stage (**c**,**d**). (**a**) Overall somatic variant detection in control (yellow) and cancer (green) patients. Dots represent the number of somatic variants reported for a sample. The detection rate is given at the top. (**b**) Same as (**a**) but according to adenocarcinoma (LUAD in dark brown) and squamous cell carcinoma (LUSC in light brown) histological subtype. (**c**) Variant allele fractions and detection rate according to tumor size, and (**d**) disease stage.

However, the variant reports still included synonymous and, based on their AF, likely germline variants (Figure 1). In addition, the population database ExAC, only containing exome variant data, and not gnomAD, additionally providing genome sequencing data, was used to filter out common germline variants. Moreover, recent studies [14,16] have shown that variants with AF < 1%, are commonly detected in healthy cfDNA samples (Figure 1). Removing all these variants (custom filtering 1–4), led to somatic variant detection in 23.5% (12/51) of patients and in only one of the 12 control (Co09) cfDNA samples (Figure 2a and Table 2 (patients) and Table S8 (controls)). The latter concerned a *BPIFB4* c.577G > A (p.(Gly193Arg)) missense variant at 2.04% AF, previously observed in a patient with melanoma (TCGA data) and here in a control patient diagnosed with mucormycosis (Supplementary Tables S1 and S8). Unfortunately, no follow-up data for this patient could be obtained.

Further characterizing the patient samples, a variant of interest could be detected in 21.9% (7/32) of patients with adenocarcinoma (LUAD) and 26.3% (5/19) of patients with squamous cell carcinoma (LUSC) (Figure 2b). All patients presented with a history of smoking, except two patients for whom this information was not available. The pooled variant list was, however, too small to investigate ageing- or tobacco-associated COSMIC signatures. As regard to tumor and overall disease stage, variant identification was possible in 50% (4/8) of pT1b, 20% (1/5) of pT1c, 18.2% (2/11) of pT2a, 33.3% (2/6) of pT2b and 60% (3/5) of pT4 tumors (Figure 2c), and in 20% (5/25), 16.7% (2/12), 30.8% (4/13) and 100% (1/1) of stage I, II, III and IV diseases (Figure 2d), respectively. Evaluating the correlation with the patient’s disease course, variant detection was not prognostic (not shown). In only three patients, disease recurred within a year; in two patients disease progressed locally (C11 and C47), and one patient developed distant metastasis (C02). One of the patients with locally progressive disease did not survive (C11). Six patients had a stable oncological status (C25, C32, C33, C45, C46 and C48). For three patients, follow-up data was not available (C13, C38 and C39; Supplementary Table S1).

**Figure 3:**
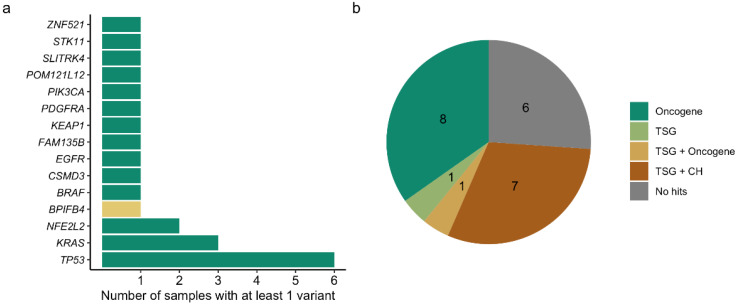
The majority of the somatic variant calls are situated in cancer driver genes. (**a**) Genes with at least one variant reported in control (yellow) and cancer (green) patients’ samples. (**b**) Number of variants reported in a gene with tumor suppressor (TSG) or oncogene prediction or associated with clonal hematopoiesis (CH). Different colors are used for different (combinations of) predictions.

The majority of positive patient samples (58.3% or 7/12) contained only one variant, followed by three samples with three variants; one sample showed two and one sample showed five variants (Figure 2a). The number of detected variants did not correlate with any variable (not shown). Variant allele fractions ranged from 1.19% to 29.47%, with a median AF of 3.65% and did not correlate with disease stage (Figure 2d). *TP53* was the most commonly altered gene, with one variant in five samples and two variants in another patient. In addition, variants in *KRAS* and *NFE2L2* were observed more than once, in three and two samples, respectively (Figure 3a). The variants in *KRAS* and *NFE2L2* occurred in the same exon but not position (Table 2). The other variants (47.8% or 11/23) concerned a single gene call (Figure 3a). Most of the variant calls (73.9% or 17/23) were situated in cancer driver genes identified by Bailey et al. [17], of which 39.1% (9/23) and 34.8% (8/23) were located in a gene with tumor suppressor or oncogene prediction, respectively (Figure 3b). There were no variant calls in genes canonically associated with clonal hematopoiesis, other than *TP53*.

**Figure 4:**
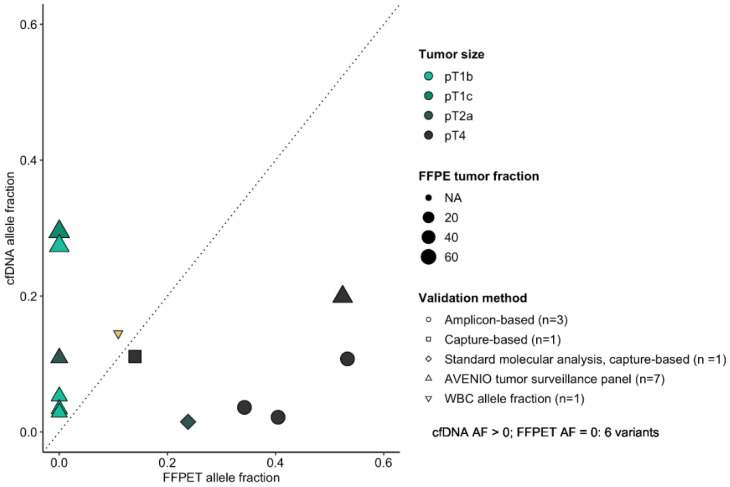
Tumor histology, tumor heterogeneity, and a low circulating tumor DNA fraction probably play a role in the discordance between cell-free DNA and tissue results. This plot represents the validation data on formalin-fixed, paraffin-embedded tumor (FFPET) tissue for 12 cell-free DNA (cfDNA) variants. The cfDNA variant allele fractions are plotted against the FFPET variant allele fractions. Symbols indicate the used validation method, whereby the size and color of the symbols annotate tumor cell percentage (FFPE tumor percentage, determined on a hematoxylin-eosin stained FFPE section) and size, respectively. For each method, the number of analyzed variants is given. Six cfDNA variants were not present in the FFPET sample. NA: not available.

For 12/23 variants (eight patients), validation data on FFPET tissue was available and six variants (all variants for four patients) were confirmed. The confirmed cfDNA variant AFs ranged from 1.48% to 19.92% and corresponded with a FFPET variant AF of 23.82% and 52.41%, respectively. However, there was no correlation between the variant AFs in both sample types, but three of the four patients did have a pT4 tumor. The cfDNA variant AFs not present in the FFPET sample ranged from 2.87% to 29.47% and corresponded with a FFPE sample tumor cell percentage of 30% to 70% (Figure 4).

Two possible canonical clonal hematopoiesis-derived variants (both in *TP53*) were checked in the matching gDNA sample (C11 and C33) and the one present at 10.91% AF in the cfDNA sample and absent in the FFPET sample, could be confirmed as a clonal hematopoiesis-derived variant at 14.49% AF (C33) (Figure 4). The other variant was present at 11.10% and 14% AF in the liquid and tumor biopsy, respectively (C11).

Standard molecular analysis on the FFPE surgical resection identified an *EGFR* (C01) or a *KRAS* (C13, C14 and C32) mutation for four patients. Tumor-variant AFs ranged from 21% to 53%. Only for patient C32, the *KRAS* mutation with a tumor AF of 23.82% was detected in the cfDNA sample at 1.48% AF (Figure 4).

For 17 patient samples without a cfDNA variant using custom filtering, one to nine (in total 48) tissue variants were detected. Variant AFs ranged from 4.09% to 51.04%, with a median variant AF of 16.60% (Supplementary Table S9).

## 4. Discussion

In this study we aimed to evaluate whether targeted deep sequencing of cfDNA would be a worthy alternative for tissue biopsy for the detection of early-stage NSCLC in patients with a lung lesion on imaging. This approach would be particularly relevant for lung lesions that appear suspect for malignancy on imaging, but are difficult to reach for a tissue-based diagnosis. Therefore, we analyzed pre-operative liquid biopsies from a cohort of 51 operable lung carcinoma patients and compared the sequencing data with 12 control patients with a resected non-malignant lung lesion.

In concordance with other studies of cfDNA alterations in healthy individuals [14,16], several low-frequency variants were detected in our control group of patients with a non-malignant lung lesion. As such, the limit of detection was set at 1% variant AF. This resulted in somatic variant detection in 23.5% (12/51) of early-stage NSCLC patients and in one of the twelve control patients. This detection rate is in line with the detection rate of 19.2% (34/177) reported in the DYNAMIC study [11], investigating perioperative changes in patients with early-stage lung cancer, using a multiplex inverse PCR technology enabling molecular bar coding and ultra-deep sequencing, combined with sequencing of a reference WBC-derived gDNA sample, to lower false-positive results due to clonal hematopoiesis. A limitation of ultrasensitive sequencing is the possibility to detect biological background mutations in cfDNA that are derived from clonal hematopoiesis, rendering a false positive result that can be eliminated by combining cfDNA and WBC analysis. For one patient in our study (C33), a somatic variant detected in *TP53* at a cfDNA variant AF > 1%, was proven to be WBC-derived, by analysis of the corresponding gDNA sample and therefore could be attributed to clonal hematopoiesis. This resulted in a sensitivity of 16% (4/25) for stage I, 16.7% (2/12) for stage II, 30.8% (4/13) for stage III, and 100% (1/1) for stage IV disease or an overall sensitivity of 21.6% and specificity of 91%. The average variant AF in the DYNAMIC study was 2.72% and comparable with the median AF of 3.65% in our study.

For only 33.3% (4/12) of patients, variants could be confirmed by analysis of malignant tissue. Tumor histology, tumor heterogeneity, and a low ctDNA fraction probably played a role in the discordance between cfDNA and tissue results, similar to other studies [18,19].

The majority of cfDNA variants in our NSCLC patient group concerned cancer driver genes. *TP53* and *KRAS* were the most commonly altered genes, not surprisingly, as there is an association between smoking and the presence of *TP53* and *KRAS* mutations in NSCLC [20,21] and our study population was enriched for (active) smokers.

We found no correlations between tumor size and stage, and variant allele frequencies, but our study population existed of operable NSCLC cancer and thus comprised mainly low-stage cancers. Studies [16,22] including a wider variation of stages do find a correlation between variant AFs and tumor stage. Some molecular tests are very sensitive to detect ctDNA, e.g., digital droplet PCR and BEAMing (beads, emulsion, amplification and magnetics) [23,24], but they require prior knowledge of the presence of specific mutations in the tumor, which makes them suitable for patient follow-up, minimal residual disease detection and resistance to targeted therapy monitoring, but precludes them for screening or diagnostic testing for early-stage NSCLC detection. The results from our study to test a ctDNA analysis-based liquid biopsy for diagnosis of suspected lung lesions on imaging demonstrated two major problems: first, variant AFs in ctDNA from early-stage carcinomas are very low (or undetectable) and, secondly, this has to be balanced against the presence of a biological background of somatic variants and sequencing errors. This pushes even ultrasensitive cfDNA analysis methods to their limits. The same difficulties are encountered in the pursuit of a screening test for non-invasive early-stage lung cancer detection, e.g., for people at risk because of a smoking history [11,12,14,25].

In their study about early-stage lung cancer, Abbosh et al. [25] estimated that a tumor volume of 10 cm^3^ (matching a lesion diameter of 2.7 cm) would result in a mean plasma variant AF of 0.1%, whereas a tumor volume of 1 cm^3^ would result in a mean variant AF of only 0.008%. It has been calculated by Fiala and Diamandis [26] that, when the fraction of ctDNA drops below 0.01%, corresponding to a tumor diameter of less than 1 cm, a volume of 10 mL of blood will probably not contain a single cancer genome, which will make the diagnosis of cancer almost impossible and prone to sampling error. Analysis of ctDNA derived from other carcinomas, e.g., breast [27] and prostate cancer [28], experiences the same problems. ctDNA analysis for early breast cancer detection experiences a sensitivity of only 10% for detecting breast cancer in asymptomatic persons in whom breast cancer has been detected by means of a screening mammography [27]. This problem of low or undetectable variant AFs in cfDNA is illustrated by the fact that, in our study, for four patients with a known *EGFR* or *KRAS* mutation in the tissue biopsy, only one variant was detectable in cfDNA by using CAPP-sequencing, and that for 17 patients in whom no cfDNA variant was detected, one to nine (in total 48) tissue variants were detected by using Roche’s AVENIO Tumor Tissue Surveillance assay. In addition to tumor diameter and load, proliferation rate and the presence of necrosis are important in predicting the success of a ctDNA analysis-based liquid biopsy for cancer detection. In the present study, we could not find a statistical difference in positivity rate between LUAD and LUSC (7/32 LUAD cases and 5/19 LUSC cases transcended the variant AF cut-off of 1%), but other studies and an earlier study of our own, also including higher stage lung cancers, report higher levels of ctDNA for LUSC [8,25]. Necrosis is more present in LUSC, especially in larger tumors, whereas mainly well-differentiated LUADs are characterized by low proliferation rates and low cell turnover [25,29]. Most NSCLCs show lower mitotic rates and Ki67 proliferation rates than, for instance, aggressive lymphomas, like diffuse large B-cell lymphoma and Hodgkin lymphoma, or small cell lung cancer. For the latter cancers, developing a liquid biopsy with a high sensitivity and specificity has been proven possible for cancer differential diagnosis without the need of biopsy, even at low clinical stages [8,30,31,32,33].

The problem of clonal hematopoiesis as a confounding factor for the development of a liquid biopsy for detection of early-stage lung cancer has been well addressed by Chabon et al. [14], who used, as in our study, CAPP-seq. They found that the majority of somatic mutations in cfDNA from lung cancer patients are derived from clonal hematopoiesis and are non-recurrent. They demonstrated that compared with tumor-derived variant alleles, clonal hematopoiesis-variant alleles occur on longer cfDNA fragments and lack tobacco smoking signatures. They integrated these findings with other molecular features in a machine-learning method, which they named ‘lung cancer likelihood in plasma’ (Lung-CLIP), to discriminate early-stage lung cancer from risk-matched controls. At 98% specificity, Lung-CLiP reached sensitivities of 41% in patients with stage I disease, but on their validation cohort of early-stage lung cancer patients, sensitivity was slightly lower than 33% (judged from their extended data).

The results of the DYNAMIC study [11] and the study of Chabon et al. [14] are in agreement with our own results and suggest that sensitivity is a major hurdle to overcome for the development of a cfDNA-based diagnostic or screening test for early-stage NSCLC. Some studies report slightly higher sensitivities. Ohara et al. [34], studying the prognostic implications of preoperative versus postoperative ctDNA with CAPP-seq on a small series of 20 patients, report a sensitivity of 40%, but patient stages were higher than in our study (stage IIa to IIIa) and their series also included a case of small cell lung cancer. Guo et al. [10], investigating the changes in ctDNA after surgical tumor resection with a targeted sequencing panel including mutational hotspots of 50 genes, report a sensitivity of 46.3 % on a series of 41 NSCLC patients, but their approach was tumor-informed, which means that they included sequencing information of tissue biopsies in their analysis.

## 5. Conclusions

To conclude, despite technical improvements (molecular and bioinformatical) for the use of liquid biopsy-based cfDNA analysis, its application for the detection of early-stage lung cancer is currently limited by sensitivity and a biological background of somatic variants. Future in-house research will focus on assays that examine multiple target loci (e.g., methylation sites) simultaneously, rather than a single mutation or a limited number of mutations. cfDNA methylation profiling by using cell-free reduced-representation bisulfite sequencing is a molecular method that has potential in that respect [35,36].

## Figures and Tables

**Table 1:** Study population’s main clinical characteristics.

Parameter	Patients		Controls	
	*n* = 51		*n* = 12	
Age (yrs)	66	(36–83)	64	(57–74)
Smoking				
Yes	44	(86.3%)	9	(75.0%)
No	3	(5.9%)	2	(16.7%)
NA	4	(7.8%)	1	(8.3%)
Histology				
LUAD	32	(62.7%)	-	-
LUSC	19	(37.3%)	-	-
T stage				
T1mi	3	(5.9%)	-	-
T1a	6	(11.8%)	-	-
T1b	8	(15.7%)	-	-
T1c	5	(9.8%)	-	-
T2a	11	(21.6%)	-	-
T2b	6	(11.8%)	-	-
T3	7	(13.7%)	-	-
T4	5	(9.8%)	-	-
Overall stage				
I	25	(49.0%)	-	-
II	12	(23.5%)	-	-
III	13	(25.5%)	-	-
IV	1	(2.0%)	-	-

**Table 2:** Variant metrics of patient samples, custom filtering.

Sample	Gene	Coding Change	Amino Acid Change	AF	FFPET AF	WBC AF	FFPET TC%	Validation Method	CNV Score
C02	*KRAS*	c.37G>T	p.Gly13Cys	2.16	40.47	N/A	50	Amplicon-based	N/A
C11	*TP53*	c.833C>T	p.Pro278Leu	11.10	14.00	Absent	50	Capture-based	N/A
C11	*NFE2L2*	c.77A>T	p.Gln26Leu	10.75	53.30	N/A	50	Amplicon-based	N/A
C11	*SLITRK4*	c.1960G>A	p.Glu654Lys	3.61	34.25	N/A	50	Amplicon-based	N/A
C13	*BRAF*	c.1781A>C	p.Asp594Ala	29.47	Absent	N/A	70	AVENIO tumor surveillance panel	N/A
C25	*KRAS*	c.35G>A	p.Gly12Asp	5.25	Absent	N/A	30	AVENIO tumor surveillance panel	N/A
C25	*KEAP1*	c.463G>T	p.Val155Phe	3.38	Absent	N/A	30	AVENIO tumor surveillance panel	N/A
C25	*STK11*	c.725G>T	p.Gly242Val	2.87	Absent	N/A	30	AVENIO tumor surveillance panel	N/A
C32	*KRAS*	c.34G>T	p.Gly12Cys	1.48	23.82	N/A	32	Capture-based	N/A
C32	*ZNF521*	c.1544G>T	p.Cys515Phe	3.68	N/A	N/A	N/A	Not validated	N/A
C33	*TP53*	c.722C>G	p.Ser241Cys	10.91	Absent	14.49	35	AVENIO tumor surveillance panel	N/A
C38	*TP53*	c.493C>T	p.Gln165*	19.92	52.41	N/A	60	AVENIO tumor surveillance panel	N/A
C39	*PDGFRA*	c.248C>T	p.Thr83Met	27.38	Absent	N/A	60	AVENIO tumor surveillance panel	N/A
C45	*TP53*	c.747G>T	p.Arg249Ser	1.19	N/A	N/A	N/A	Not validated	N/A
C45	*CSMD3*	c.3406C>A	p.Leu1136Met	1.85	N/A	N/A	N/A	Not validated	N/A
C45	*FAM135B*	c.1347G>T	p.Met449Ile	1.25	N/A	N/A	N/A	Not validated	N/A
C46	*TP53*	c.844C>T	p.Arg282Trp	5.28	N/A	N/A	N/A	Not validated	N/A
C46	*TP53*	c.746G>T	p.Arg249Met	1.81	N/A	N/A	N/A	Not validated	N/A
C46	*PIK3CA*	c.1624G>A	p.Glu542Lys	3.22	N/A	N/A	N/A	Not validated	N/A
C46	*EGFR*	N/A	N/A	N/A	N/A	N/A	N/A	Not validated	6.47
C46	*POM121L12*	c.325G>T	p.Gly109Trp	3.95	N/A	N/A	N/A	Not validated	N/A
C47	*TP53*	c.376-1G>A	N/A	2.29	N/A	N/A	N/A	Not validated	N/A
C48	*NFE2L2*	c.100C>G	p.Arg34Gly	14.65	N/A	N/A	N/A	Not validated	N/A

AF: allele frequency; FFPET: formalin-fixed, paraffin-embedded tumor; WBC: white blood cell; TC: tumor cell; CNV: copy number variation; N/A: not applicable.

## Data Availability

The data presented in this study are available in Supplementary Tables S3–S9.

## Supplementary Materials

The following supporting information can be downloaded at: https://www.mdpi.com/article/10.3390/cancers14082031/s1, Table S1: Study population’s clinical characteristics; Table S2: Standard molecular analysis results; Table S3: Sample metrics of patient samples; Table S4: Sample metrics of control samples; Table S5: Sample metrics of tissue samples; Table S6: Variant metrics of patient samples, default filtering; Table S7: Variant metrics of control samples, default filtering; Table S8: Variant metrics of control samples, custom filtering; Table S9: Variant metrics of the AVENIO tumor tissue surveillance assay, custom filtering.
